# Stairway to memory: Left-hemispheric alpha dynamics index the progressive loading of items into a short-term store

**DOI:** 10.1016/j.neuroimage.2021.118024

**Published:** 2021-04-06

**Authors:** Alex I. Wiesman, Nicholas J. Christopher-Hayes, Tony W. Wilson

**Affiliations:** a College of Medicine, University of Nebraska Medical Center, Omaha 68198-8422, NE, United States; b Institute for Human Neuroscience, Boys Town National Research Hospital, Omaha, NE, United States

**Keywords:** Short-term memory, Encoding, Load effects, Sequence memory, Neural oscillations

## Abstract

The encoding, maintenance, and subsequent retrieval of memories over short time intervals is an essential cognitive function. Load effects on the neural dynamics supporting the maintenance of short-term memories have been well studied, but experimental design limitations have hindered the study of similar effects during the encoding of information into online memory stores. Theoretically, the active encoding of complex visual stimuli into memory must also recruit neural resources in a manner that scales with memory load. Understanding the neural systems supporting this encoding load effect is of particular importance, as some patient populations exhibit difficulties specifically with the encoding, and not the maintenance, of short-term memories. Using magnetoencephalography, a visual sequence memory paradigm, and a novel encoding slope analysis, we provide evidence for a left-lateralized network of regions, oscillating in the alpha frequency range, that exhibit a progressive loading effect of complex visual stimulus information during memory encoding. This progressive encoding load effect significantly tracked the eventual retrieval of item-order memories at the single trial level, and neural activity in these regions was functionally dissociated from that of earlier visual networks. These findings suggest that the active encoding of stimulus information into short-term stores recruits a left-lateralized network of frontal, parietal, and temporal regions, and might be susceptible to modulation (e.g., using non-invasive stimulation) in the alpha band.

## 1. Introduction

Short-term memory is a dynamic process, requiring the encoding of contextual and temporal associations amongst complex stimuli, as well as the subsequent maintenance of this information. The effective loading of these stimuli into a short-term cognitive “store” during memory encoding is imperative to everyday function, and requires a high-fidelity representation of both item features and the temporal order of their occurrence. Numerous studies have investigated the effects of memory load on the neural dynamics supporting short-term memory maintenance, and found that neural activity scales with increased amounts of information to be remembered (Busch and Herrmann, 2003; Jackson et al., 2008; Jensen et al., 2002; Leung et al., 2004; Manoach et al., 1997; Proskovec et al., 2019a, 2019b; Raabe et al., 2013; Sauseng et al., 2009; Tomasi et al., 2007; Tuladhar et al., 2007). However, despite extensive literature focusing on the neural underpinnings of such load effects during memory maintenance, comparatively little is known regarding how similar differences in memory load might be represented during the active encoding of new information. It is likely that this gap in knowledge is at least partially due to an inherent disparity in the amount of visual information presented to participants in the different load conditions in traditional short-term memory paradigms. This knowledge, however, is essential, as it has been suggested that some patient populations exhibit difficulties specifically with the encoding, and not the maintenance, of item information in the short-term (e.g., Golby et al., 2005; Kim et al., 2014; Lenartowicz et al., 2019; Rombouts et al., 2000; Wiesman et al., 2016). Thus, a paradigm and analysis pipeline with which to study the active loading of stimulus information into short-term memory stores in health and pathological conditions is needed.

A number of theoretical approaches have been used to study the neural systems supporting short-term memory in humans, and in particular, oscillatory patterns of population-level neural activity have been established as being essential to this cognitive function (Başar et al., 2000; Heinrichs-Graham and Wilson, 2015; Jensen et al., 2002, 2007; Jensen and Tesche, 2002; Klimesch, 1999). Alpha-frequency activity has been of particular interest in this line of research, as within occipital cortices this oscillatory rhythm has been shown to scale as a function of load during the maintenance of short-term memories (Fukuda et al., 2015; Jensen et al., 2002; Kardan et al., 2020; Manza et al., 2014; Proskovec et al., 2019a, 2019b; Roux et al., 2012; Tuladhar et al., 2007), and to increase when distractors are present during such maintenance (Bonnefond and Jensen, 2012). Despite an ongoing debate over whether this alpha response reflects distractor suppression or target enhancement (Foster and Awh, 2018; Wöstmann et al., 2019; Zhigalov and Jensen, 2020), it is clear that alpha oscillations are particularly salient when considering short-term memory load effects. While theta and gamma oscillations are also thought to be essential to short-term memory processing, far less data indicates that neural responses in these frequencies scale as a function of memory load. Instead, studies have suggested that hippocampal theta and gamma oscillations phasically organize stimulus information during short-term memory processing (Lisman, 2010). In contrast, theta responses in occipital cortices have previously been shown to index temporal expectation and initial alerting to expected visual stimuli (Kayser et al., 2012; Makeig et al., 2002; Ronconi et al., 2017; Wiesman et al., 2017; Wiesman and Wilson, 2019), while gamma responses in early visual cortices are sensitive to manipulations of a number of discrete stimulus features (Bertrand and Tallon-Baudry, 2000; Busch et al., 2004; Edden et al., 2009; Muthukumaraswamy and Singh, 2013; Siegel et al., 2007; Swettenham et al., 2009; Vidal et al., 2006; Womelsdorf et al., 2006). These findings have substantially advanced our understanding of the spectral and spatial neural dynamics serving short-term memory, however, an important knowledge gap exists regarding what oscillatory rhythms and regions of the cortex display similar load effects while items are actively being encoded.

**Fig. 1.**
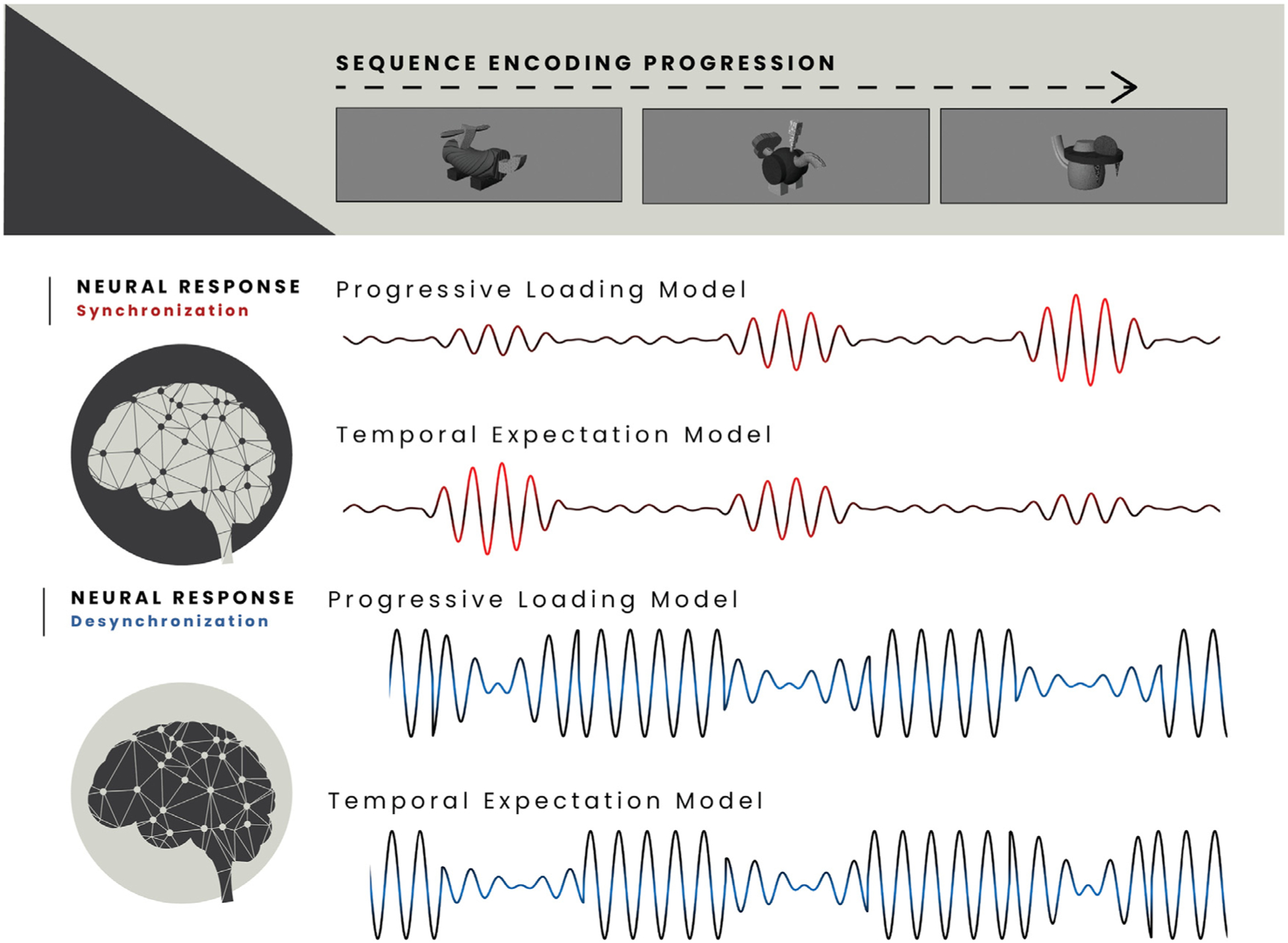
Competing neural response models for sequential stimuli. Expected patterns of population-level neural responses (modulated sinusoids) to sequentially presented complex visual stimuli (top), based on two competing hypothesized models. When the underlying neural response is a synchronization from baseline (middle; red), responses in brain regions *progressively loading* stimulus information into a memory store would be expected to exhibit a linear increase in relative amplitude, while those indexing temporal expectation and/or attentional alerting to the onset of the sequence would be expected to exhibit a decrease in amplitude. Conversely, in regions where the neural response is a decrease in synchrony from baseline (bottom; blue), regions indexing load effects would exhibit a linear decrease in relative amplitude, while those indexing attention/expectation would exhibit a linear increase.

Herein, we characterize, both spatially and spectrally, the neural activity serving the active loading of item-order information into short-term memory stores. Towards this goal, we use the high spatio-temporal precision of magnetoencephalography (MEG) and a sequence memory task that enables us to model increases in neural response amplitude as a function of increasing memory load within each participant at the level of the single trial. Given previous well-replicated findings of a positive linear relationship between memory load and neural response amplitude during maintenance, we hypothesized that a distributed set of cortical regions would exhibit significant load effects during short-term memory encoding (i.e., a *progressive loading model*; Fig. 1). Due to the load-dependent importance of alpha-frequency neural activity in the processing and storage of short-term memories in different contexts, we expected that alpha band responses would, in particular, be central to such active encoding load effects. Further, if these regions involved in encoding load effects were indeed performing a function separate from occipital cortices oscillating at a similar frequency, then neural activity in these regions should exhibit temporary decreases in connectivity from earlier visual networks to preserve the fidelity of local stimulus representations. In contrast, since theta band responses in occipital cortices are well-supported as indexing temporal expectation and initial alerting to expected visual stimuli, we anticipated that these responses would decrease as a function of sequence memory progression (i.e., a *temporal expectation model* ; Fig. 1). We did not have strong predictions regarding activity in the gamma band.

## 2. Materials and methods

### 2.1. Participants

We enrolled 23 healthy young adults (mean age = 26.09 years; SD = 3.87 years; range = 20–33 years; 16 males/7 females; 21 right-handed) for participation in this study. Exclusionary criteria included any medical illness affecting CNS function, any neurological disorder, history of head trauma, any non-removable metal implant that would adversely affect data acquisition, current substance abuse, and poor performance (i.e., accuracy *<* [mean - 3 SD]; 2 participants excluded using this criteria) on the memory task. The Institutional Review Board at the University of Nebraska Medical Center reviewed and approved this investigation. After complete description of the study, written informed consent was acquired from each participant. All participants had normal or corrected-to-normal vision. All participants completed the same experimental protocol.

### 2.2. Sequence memory paradigm

**Fig. 2.**
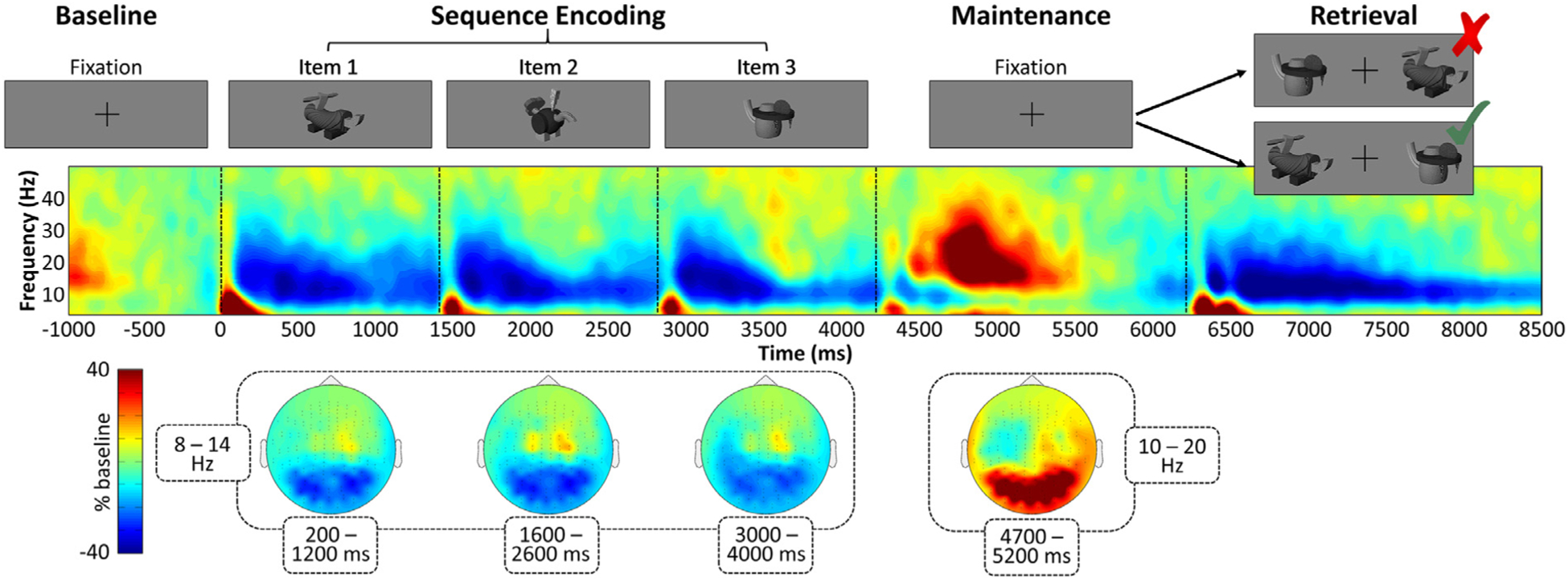
Experimental paradigm and neural oscillatory responses. The sequence memory task (top) required participants to hold the identity and temporal order of three visually complex novel stimuli in memory, and then respond as to whether the order of two target stimuli (from left to right) did or did not match the order of the original sequence. Oscillatory neural responses to the memory task are displayed from a representative sensor (middle; MEG1923). Significant responses from baseline in the alpha band are plotted topographically (bottom) for each of the three sequence stimuli (8–14 Hz), as well as for the maintenance period (10–20 Hz). Participants exhibited robust neural oscillatory responses (bottom) to the encoded stimuli in the alpha band during encoding (8–14 Hz) and maintenance (10–20 Hz). The shared colorbar indicates percent change from baseline for both the spectrogram and topographic data. See also Figures S1 and S2 for representative spectrograms and topographic plots for the theta and gamma responses, respectively.

We developed a new visual short-term memory paradigm for this study, requiring participants to remember a sequence of novel, visually complex items (Fig. 2, top). Each trial began with a central fixation presented for a randomly varied inter-stimulus interval of 2000–2400 ms. This fixation was then replaced by a sequence of three visual stimuli (item presentation duration: 1400 ms; total presentation time: 4200 ms), the item-order information of which would subsequently be tested. Each of the three items was a novel stimulus modeled to simulate the complexity and properties of real-world stimuli (Barry et al., 2014). To facilitate later recognition, the three items within each trial were selected from three separate “families” of these stimuli, where different members of the same family share an underlying “body-shape”, but have distinctive and unique combinations of peripheral components. Importantly, the order of presentation of stimuli from each of the three families was randomized over the course of the task, which ensured balancing of all visual features across the three encoded items. After this *encoding* period was complete, the third sequence-item disappeared, and the central fixation reappeared for another 2000 ms (*maintenance*), after which two of the previously-seen encoded items appeared for 2300 ms (*retrieval*). During retrieval, two of the items from the encoding set were presented equidistant on either side of the fixation. Participants were instructed to respond at this stage, as to whether the item on the left had appeared earlier in the sequence than the item on the right (*preserved* trials; right-handed index finger button press), or if the temporal order of the items had been “switched” (*reordered* trials; right-handed middle finger button press). All participants were instructed to respond as quickly and accurately as possible, and only correct trials were included in further analyses. Preserved and reordered trial types were pseudo-randomized over the course of the experiment, such that neither condition was repeated more than twice in a row. Participants each performed 120 trials of the task (60 preserved and 60 reordered), for a total recording time of ~22 min. The custom experimental paradigm was programmed in Matlab (Mathworks, Inc., Massachusetts, USA) using *Psychophysics Toolbox Version 3* (Brainard, 1997) and back-projected onto a semi-translucent non-ferromagnetic screen at an approximate distance of 1.07 m, using a Panasonic PT-D7700U-K model DLP projector with a refresh rate of 60 Hz and a contrast ratio of 4000:1. Each encoded visual item subtended an approximate visual angle of 5.45° vertically and 6.81° horizontally.

### 2.3. MEG data acquisition

All recordings were conducted in a one-layer magnetically shielded room with active shielding engaged for environmental noise compensation. Neuromagnetic responses were sampled continuously at 1 kHz with an acquisition bandwidth of 0.1–330 Hz using a 306-sensor Elekta/MEGIN MEG system (Helsinki, Finland) equipped with 204 planar gradiometers and 102 magnetometers. Participants were monitored during data acquisition via real-time audio-video feeds from inside the shielded room. Each MEG dataset was individually corrected for head motion and subjected to noise reduction using the signal space separation method with a temporal extension (correlation limit: 0.950; correlation window duration: 6 s; Taulu and Simola, 2006). Only data from the gradiometers were used for further analysis.

### 2.4. Structural MRI processing and MEG coregistration

Preceding MEG measurement, four coils were attached to the participant’s head and localized, together with the three fiducial points and scalp surface, using a 3-D digitizer (Fastrak 3SF0002, Polhemus Navigator Sciences, Colchester, VT, USA). Once the participant was positioned for MEG recording, an electric current with a unique frequency label (i.e., 293, 307, 314, and 321 Hz) was fed to each of the coils. This induced a measurable magnetic field and allowed each coil to be localized in reference to the sensors throughout the recording session. Since coil locations were also known in head coordinates, all MEG measurements could be transformed into a common coordinate system. With this coordinate system, each participant’s MEG data were co-registered with structural T1-weighted MRI data using BESA MRI (Version 2.0) prior to source-space analysis. Structural MRI data were aligned parallel to the anterior and posterior commissures and transformed into a standard space. Following source analysis (i.e., beamforming), each participant’s 4.0 × 4.0 × 4.0 mm functional images were also transformed into the same standard space using the transform that was previously applied to the structural MRI volume.

### 2.5. MEG preprocessing, time-frequency transformation, and sensor-level statistics

Cardiac and blink artifacts were removed from the data using signal-space projection (SSP), which was subsequently accounted for during source reconstruction (Uusitalo and Ilmoniemi, 1997). The continuous magnetic time series was then filtered between 0.5 – 200 Hz plus a 60 Hz notch filter, and divided into 9500 ms epochs, with the baseline extending from 500 ms to 0 ms prior to the onset of the encoding sequence stimuli. Epochs containing artifacts were rejected using a fixed threshold method, supplemented with visual inspection. Briefly, in MEG, the raw signal amplitude is strongly affected by the distance between the brain and the MEG sensor array, as the magnetic field strength falls off sharply as the distance from the current source increases. To account for this source of variance across participants, as well as actual variance in neural response amplitude, we used an individually-determined threshold based on the signal distribution for both signal amplitude and gradient to reject artifacts. Across all participants, the average amplitude threshold was 1054.76 (SD = 186.52) fT/cm and the average gradient threshold was 192.14 (SD = 140.96) fT/(cm ∂T). Across participants, an average of 93.43 (SD = 8.09) trials were used for further analysis.

To investigate the oscillatory responses associated with processing of the visually-complex sequence stimuli, we next transformed the same post-artifact-rejection epochs into the time-frequency domain using complex demodulation (Hoechstetter et al., 2004; Kovach and Gander, 2016; Papp and Ktonas, 1977). Briefly, complex demodulation works by first transforming the signal into the frequency space, using a Fast Fourier Transform (FFT). This results in a frequency spectrum, inherently containing the same power and cross spectrum information as the original signal. From here, this frequency spectrum is (de)modulated in a step-wise manner to adopt the center frequency of a series of complex sinusoids with increasing carrier frequencies, in a process termed “heterodyning.” These resulting signals are then low-pass filtered to reduce spectral leakage, and thus the nature of this filter inherently determines the time and frequency resolution of the resulting data. For this study, the time-frequency analysis was performed with the following parameters: frequency-step of 2 Hz; time-step of 25 ms; frequency range of 4 – 100 Hz; using a 4 Hz lowpass finite impulse response (FIR) filter with a full-width half maximum (FWHM) in the time domain of ~115 ms. The resulting spectral power estimations per sensor were averaged over trials to generate time-frequency plots of mean spectral density, which were normalized by the baseline power of each respective bin, calculated as the mean power during the −500 to 0 ms time period. The time-frequency windows used for the source analysis were determined by means of a paired-sample cluster-based permutation test against baseline across all participants and the entire frequency range (4 – 100 Hz), with an initial cluster threshold of *p <* 0.05, a final cluster significance threshold of *p <* 0.001, and 1000 permutations.

### 2.6. MEG source imaging

Time-frequency resolved beamformer source images were computed using the dynamic imaging of coherent sources (DICS; regularization: singular value decomposition; Gross et al., 2001) approach, which uses the time-frequency averaged cross-spectral density to calculate voxel-wise estimates of neural power and/or coherence. Source images were regularized using the truncated singular value decomposition approach, as implemented in BESA Research 6.1, such that all singular values lower than 0.0001% of the maximum singular value were set to zero. Following convention, we computed noise-normalized, source power per voxel in each participant using active (i.e., task) and passive (i.e., baseline) periods of equal duration and bandwidth. The use of active and passive periods with comparable durations and bandwidths is essential, as it ensures that the dual-state beamformer is not biased by the inclusion of different amounts of data in the computation of one versus the other. Such images are typically referred to as pseudo-t maps, with units (pseudo-t) that reflect noise-normalized power differences (i.e., active vs. passive) per voxel. This approach generated three-dimensional participant-level pseudo-t maps per each of the three encoding stimuli, for each time-frequency cluster identified in the sensor-level analysis (i.e., theta, alpha, and gamma).

### 2.7. Whole-brain encoding slope computation

From the whole-brain response maps that we computed per sequence item, we then computed voxel-wise estimates of the response slope across the sequence. Using the *polyfit* function in Matlab, we fit a linear model across the three neural responses (i.e., one per sequence item) at each voxel, and then extracted the slope of each of these models, resulting in a participant-level slope map per oscillatory response. Each of these whole-brain maps represented the spatially-filtered linear trend in response amplitude across the presentation of the three sequence items, per each oscillatory response, which could then be used to test our hypotheses. We tested these slope-maps voxel-wise against a null hypothesis of zero (i.e., no significant positive or negative encoding slope). Statistical testing, including multiple comparisons correction on the whole-brain images is described below in the *Statistical Analysis and Software* section.

### 2.8. Single-trial virtual sensor extraction and linear mixed effects modeling

Using the peak voxel locations identified in the whole-brain encoding slope statistics, virtual sensor data were computed by applying the sensor-weighting matrix to the preprocessed signal vector, which yielded a time series corresponding to the location of interest. These virtual sensor data were then decomposed into time-frequency space *per trial* and averaged across the previously identified time-frequency extents (i.e., 8–14 Hz, 200–1200 ms after the onset of each sequence item). This resulted in amplitude estimates of each alpha-frequency response per participant to each of the three encoding sequence items. Using these estimates, slope values were computed per participant, per trial, and used to compute a linear mixed effects model of single-trial task performance (i.e., RT) on encoding slope dynamics across the identified regions (model = RT ~ Slope[1] + Slope[2] + …Slope[*n*]; random effects = ~1|Participant/Trial).

### 2.9. Time-frequency resolved volumetric functional connectivity computation

To address hypotheses regarding connectivity in the time-frequency domain, peak voxels identified in the initial whole-brain encoding slope analysis were used as seeds for computation of whole-brain cortico-cortical coherence (again using DICS; Gross et al., 2001), reflecting time-frequency-resolved connectivity between these seeds and all other voxels in the brain (Koshy et al., 2020; Schoffelen and Gross, 2009; Wiesman and Wilson, 2020). Similar to the power analysis, coherence maps computed from active periods were normalized to coherence maps from passive periods, resulting in whole-brain estimates of percent-change in coherence from baseline for each participant, per each sequence item. Using a voxel-wise general linear model, these whole-brain cortico-cortical coherence images were tested against a null hypothesis of zero (i.e., no coherence), including the effects of amplitude at both the seed voxel and at each voxel being modeled as covariates of no interest. All reported clusters for the coherence analysis are thus significant increases/decreases in functional coherence from baseline, above and beyond the effects of any spatially co-localized changes in response amplitude from baseline.

### 2.10. Statistical analyses and software

All data preprocessing, coregistration, sensor-, and source-level analyses were performed in the Brain Electrical Source Analysis software suite (BESA Research v6.1 and BESA MRI v2.0). Cluster-based permutation testing on sensor-array data was performed in BESA Statistics (v2.0), and the single trial analysis was performed using the *nlme* package in *R* (Team, 2017). Outliers were identified post-hoc in the single-trial linear mixed-effects models using a threshold of normalized residuals ≥ the 97.5 percentile, and, when necessary, these models were re-computed without these data points to confirm initial findings. Whole-brain statistics were performed using custom functions written in Matlab 2018b (Mathworks, Inc., Massachusetts, USA). Correction for whole-brain multiple comparisons used an initial voxel-wise threshold of *p <* 0.005, and a secondary correction using either FWE cluster correction in SPM12 (whole-brain slope models) or a cluster extent correction of *k >* 500 voxels (one-sample coherence tests with voxel-wise amplitude covariates).

## 3. Results

Twenty-three healthy adults completed a memory task during the MEG recording, wherein they were required to memorize the temporal order and identity of three visually-complex, semi-naturalistic stimuli, and maintain this information for a short delay. Two of the original stimuli were then presented side-by-side, and participants were asked to respond as to whether the original presentation order of the items was intact (Fig. 2, top). Before exclusions, participants performed the task with a mean accuracy of 86.21% (SD = 9.58%) and a mean RT of 1330.79 ms (SD = 190.23 ms). Two participants were excluded due to poor performance (i.e., accuracy less than 3 SD below the mean), and the remaining participants (mean age = 26.14 years, SD = 3.90) performed well on the task (mean accuracy: 88.46% [SD = 6.22%]; mean RT: 1303.68 ms [SD = 171.62 ms]). Time-frequency analysis of the MEG data revealed multi-spectral neural responses to each encoded visual item in the theta (4–8 Hz), alpha (8–14 Hz), and gamma (64–76 Hz) bands originating from posterior occipito-temporal regions (Figs. 2 and S1–S2; *p <* 0.001, corrected). Furthermore, responses in the alpha range appeared to extend into anterior left-lateralized cortices as a function of encoding progression (Fig. 2, bottom). Each of these time-frequency responses was imaged to the level of the cortex using a frequency-resolved beamformer, and the resulting whole-brain maps were used to test our hypothesized models.

### 3.1. Spatially-distinct oscillatory networks index the progressive encoding of item and temporal features

We first aimed to determine which spectrally- and spatially-defined neural responses indexed the short-term loading of progressively increasing item-order information. Towards this goal, we fit a linear slope for each voxel per participant, using the amplitude of the spectrally defined neural responses to the three sequential stimuli. This resulted in a whole-brain map of modeled slopes per participant, per each neural oscillatory response, which represented the trend in response amplitude across the encoding of the three stimuli. For interpretation, a trend of increasing response amplitude across the three stimuli would be expected to represent a short-term memory loading effect, while the opposite trend would represent a lower-order alerting effect towards expected visual stimuli (Fig. 1). Initial one-sample t-tests of these slopes against zero indicated significant clusters in all three of the neural oscillatory responses.

**Fig. 3.**
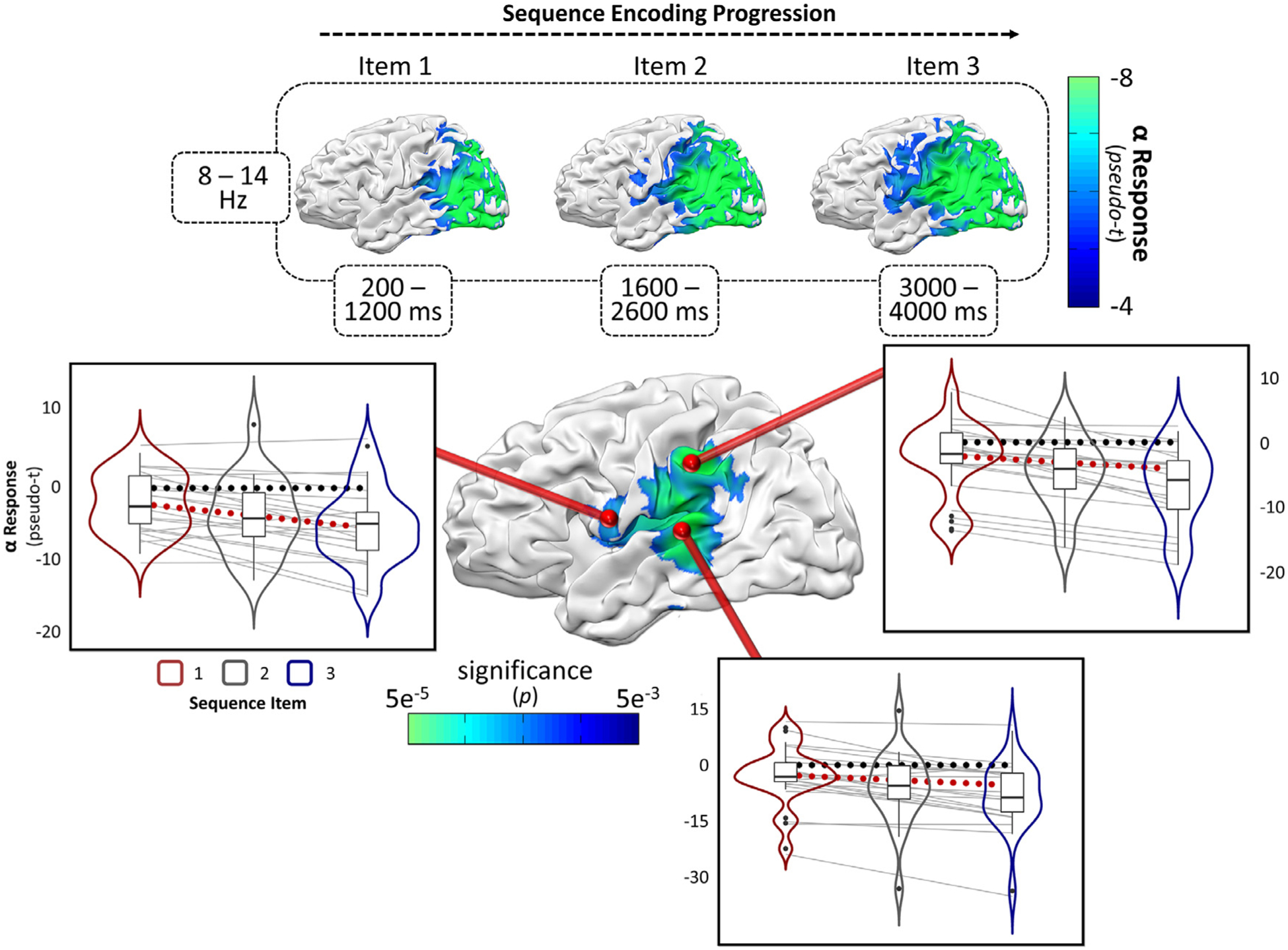
Left-hemispheric alpha oscillations index the active loading of stimulus information into short-term memory. Group-averaged alpha-frequency oscillations began in bilateral occipital cortices, and progressively recruited more anterior regions in the left hemisphere (maps above). Whole-brain encoding slope analysis revealed three left-lateralized regions that exhibited a stronger alpha desynchronization response as a function of item number in the sequence, including the inferior frontal, inferior parietal, and superior temporal cortices (center map). From each significant peak in the encoding slope analysis, each participant’s encoding slope is plotted (grey lines), along with the item means, first and third quartiles, and minima and maxima (box plots), as well as the probability density (violin plots) for each sequence item. The black dotted line indicates the null hypothesis of no slope, and the red dotted line indicates the mean slope across all participants.

In the theta band, there was a significant reduction in the neural synchronization response across the presentation of the three visual stimuli in a distributed cluster that spanned the bilateral occipital cortices (Figure S3). This indicated that posterior theta oscillations likely reflect early alerting towards temporally-expected salient stimuli, an interpretation that is supported by previous literature (Makeig et al., 2002; Wiesman et al., 2017). In contrast, the alpha response indicated support for a short-term memory load effect during encoding in a large cluster spanning left inferior frontal (IFC), inferior parietal (IPC), and superior temporal (STC) cortices (*p*_*cluster*_ = 0.0100, FWE-corrected; Fig. 3). The direction of this effect was such that the alpha desynchronization response in these regions became substantially stronger (i.e., *more negative relative to baseline*) as the sequence of encoded items progressed. Post-hoc testing of these data revealed a significant stepwise decrease in relative alpha amplitude as a function of sequence progression, which indicates that all three items were being encoded by the participants (all *p* ’s *<* 0.05, Figure S4), making alternative task strategies unlikely.

Using our initial uncorrected significance threshold (voxel-wise *p <* 0.005), gamma band activity also exhibited support for a load effect during encoding in right parieto-occipital cortex, while alpha band activity also exhibited support for an alerting effect in the left lateral occipital cortices (Figure S3). Although potentially of interest, these findings did not survive second-level correction for multiple comparisons, and we do not interpret them herein. However, our corrections for multiple comparisons could be viewed as relatively stringent, and thus while these findings warrant investigation in future studies, they should be interpreted cautiously.

### 3.2. Left-lateralized alpha encoding slopes covary with sequence memory performance and maintenance responses at the single-trial level

**Fig. 4.**
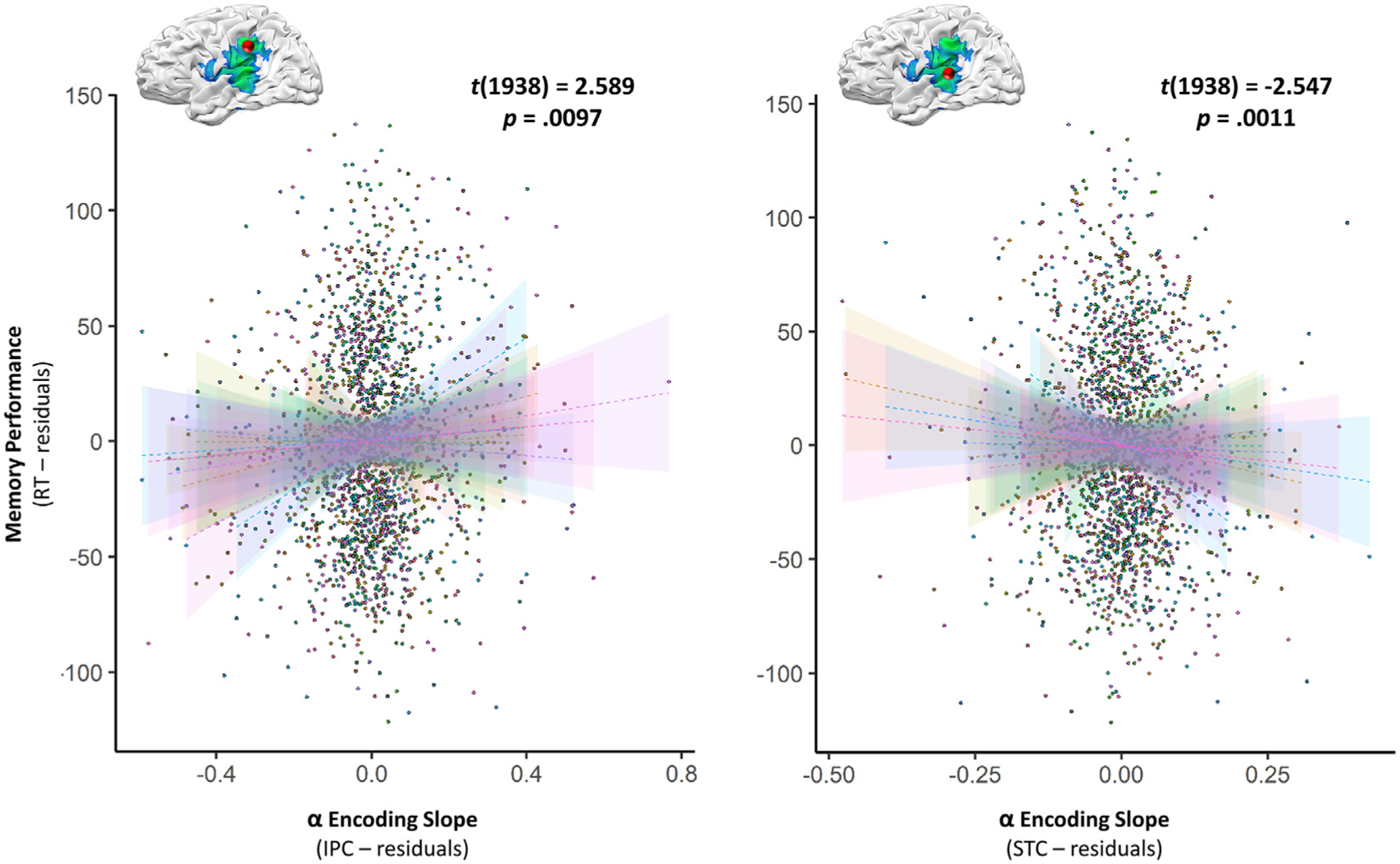
Alpha memory load dynamics during encoding covary with single-trial behavioral performance. Linear mixed effects modeling of single-trial data revealed that progressive left-hemispheric alpha dynamics had a robust impact on temporal order memory. Encoding slope residuals from the model are plotted on the *x*-axis, and memory performance residuals on the *y*-axis. Participant-level single-trial data are plotted in unique colors, with lines-of-best-fit and corresponding confidence intervals overlaid, per participant, in the same color.

To establish the relevance of this left-lateralized alpha loading effect during encoding for performance on the sequence memory task, we extracted single-trial virtual sensor data for the alpha responses from the peak voxel locations identified in the whole-brain slope analysis. We used these values to compute a neural response slope across the three items, but this time for each trial per participant. We then computed a linear mixed-effects model that related item-order memory performance (i.e., trial-wise reaction time [RT]) to these single-trial neural response slopes. The neural response slope in both the left IPC and STC was strongly related to sequence memory performance (Fig. 4; Table S1). In the IPC, this relationship was such that a more robust loading effect during encoding (i.e., a more negative slope) significantly covaried with enhanced task performance (i.e., lower reaction times) across trials (*t*(1938) = 2.59, *p* = 0.0097). In contrast, the opposite pattern of effects was observed in the STC (*t*(1938) = − 2.55, *p* = 0.0110), and the IFC neural response slope did not significantly relate to task performance (*t*(1938) = 1.09, *p* = 0.2749). No data points in this model were identified as exceeding our outlier threshold (normalized residuals ≥ 97.5 percentile).

**Fig. 5.**
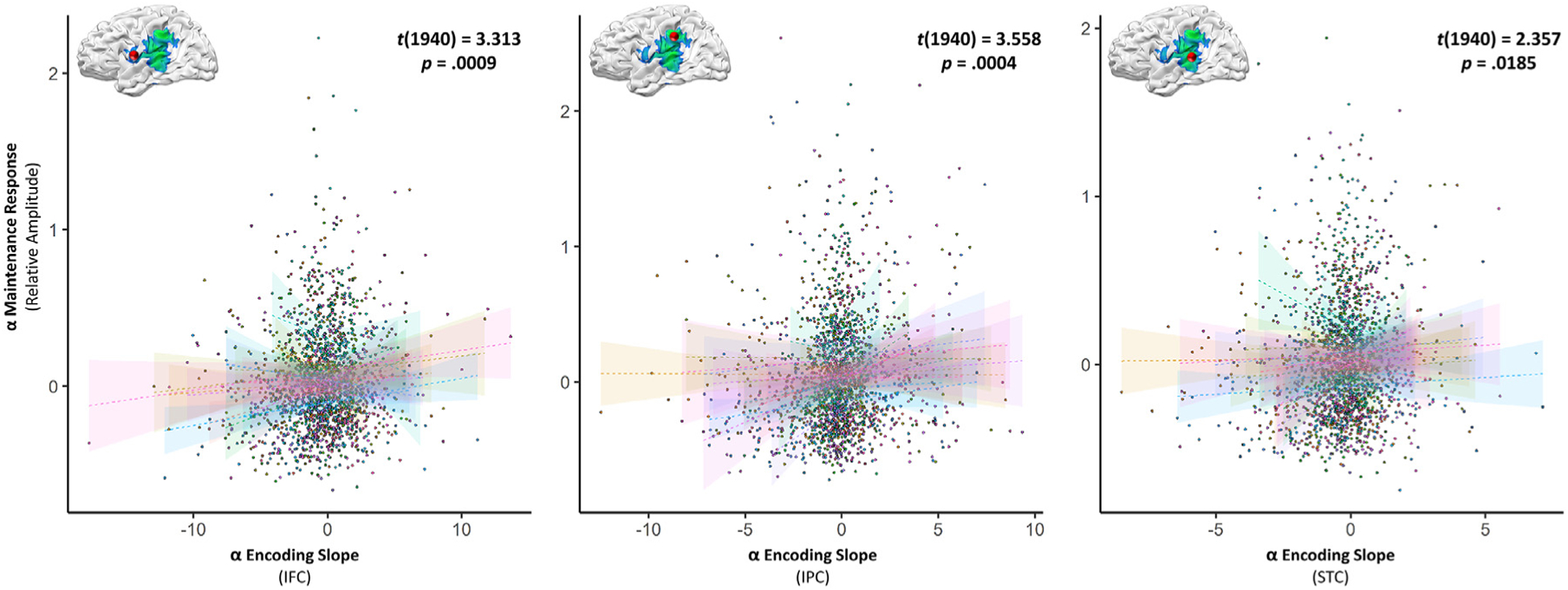
Progressive alpha encoding dynamics covary with single-trial maintenance dynamics. Linear mixed effects modeling of single-trial data revealed that progressive left-hemispheric alpha dynamics had a significant impact on subsequent neural responses during the maintenance period in the same region. Encoding slopes per region are plotted on the *x*-axis, against the alpha-frequency neural response during maintenance from the same peak voxel on the *y*-axis. Participant-level single-trial data are plotted in unique colors, with lines-of-best-fit and corresponding confidence intervals overlaid, per participant, in the same color.

The left-lateralized regions identified in the whole-brain slope analysis have also been implicated previously in the maintenance of short-term memories, and so it might be expected that more robust loading of item-order information in these regions during encoding would translate to greater recruitment of these same regions during maintenance. In other words, we would expect a more negative encoding slope to be related to a stronger suppression of alpha activity during maintenance in these regions. We tested this hypothesis using the same trial-wise slope values used for the single-trial behavior analysis, and computed a linear mixed-effects model of the relationship between the alpha response slope in each region and the subsequent neural response during maintenance at that same peak. All three regions exhibited a significant relationship between alpha encoding response slope and the maintenance response, such that a stronger loading effect (i.e., a more negative slope) was related to a stronger neural response (i.e., alpha suppression) during maintenance (IFC: *t*(1940) = 3.313, *p* = 0.0009; IPC: *t*(1940) = 3.558, *p* = 0.0004; STC: *t*(1940) = 2.357, *p* = 0.0185; Fig. 5; Tables S2–S4). After excluding data points identified as exceeding our outlier threshold (normalized residuals ≥ 97.5 percentile; IFC: 2; IPC: 1; STC: 1) and re-running these models, the initially-significant relationships were only strengthened (IFC: *t*(1938) = 3.490, *p* = 0.0005; IPC: *t*(1939) = 3.874, *p* = 0.0001; STC: *t*(1940) = 2.428, *p* = 0.0153).

### 3.3. The left-lateralized alpha memory store is functionally disconnected from early sensory cortex during item-order memory encoding

If this left-lateralized alpha memory network functions as a progressive memory store, it would also be expected to do so at least semiautonomously from early sensory processing. To explore the functional independence of this network, we used each of the three peaks identified in the neural slope analysis as seeds for whole-brain functional connectivity analysis in the alpha band. Alpha responses in all three of the left-hemispheric memory store regions (i.e., the STC, IFC, and IPC) were functionally decoupled from early visual responses during encoding of the item sequences, above and beyond the effect of response amplitude (Figure S5). In other words, connectivity between these three left hemispheric regions and sensory cortices significantly decreased during encoding relative to baseline.

## 4. Discussion

The ability to actively load stimulus information into a short-term memory store during encoding is essential to normative cognitive function. We find that such memory loading recruits neural populations across regions of the left frontal, parietal, and temporal cortices, and that this distributed processing is organized by alpha-frequency rhythmic fluctuations in neuronal activity.

Using a whole-brain encoding slope analysis, we find that alpha-frequency neural oscillations in the left hemisphere robustly index the progressive load effects during short-term memory encoding. The lateralization of this network is not surprising, given the importance of the left hemisphere for visual mental imagery (Farah et al., 1985; Marsolek, 1995), memory function (Baldo and Dronkers, 2006; Embury et al., 2019; Hayama et al., 2012; Heinrichs-Graham and Wilson, 2015; Majerus et al., 2007, 2006; Proskovec et al., 2016, 2018; Sneve et al., 2013; Wiesman et al., 2016), and temporal order information (Hayama et al., 2012; Majerus et al., 2007, 2006; Sneve et al., 2013). Further, alpha activity is known to be modulated by memory load during short-term maintenance (Fukuda et al., 2015; Jensen et al., 2002; Kardan et al., 2020; Manza et al., 2014; Proskovec et al., 2019a, 2019b; Roux et al., 2012; Tuladhar et al., 2007). Decreases from baseline levels of alpha synchrony in these association areas are widely thought to represent a departure from resting functional inhibition, in favor of more computationally expensive local processing of information (Handel et al., 2011; Jensen and Mazaheri, 2010; Klimesch, 2012; Wiesman et al., 2018a), however this theory is currently under debate (Foster and Awh, 2018; Wöstmann et al., 2019; Zhigalov and Jensen, 2020). The current study extends these findings to the encoding phase, by showing that alpha-frequency desynchronizations index the active loading of stimulus information into short-term memory stores in left-lateralized regions of the cortex.

The specific cortical regions comprising this loading effect during encoding include the IFC, IPC, and STC, all of which are well established as being essential for normal short-term and working memory function (Baldo and Dronkers, 2006; Embury et al., 2019; Hayama et al., 2012; Heinrichs-Graham and Wilson, 2015; Majerus et al., 2007, 2006; Proskovec et al., 2016, 2018; Sneve et al., 2013; Wiesman et al., 2016). Interestingly, all three of these regions are also thought to provide functionally dissociable contributions to the active encoding of verbal and visual stimuli (Baldo and Dronkers, 2006; Hayama et al., 2012; Majerus et al., 2007, 2006; Proskovec et al., 2018; Sneve et al., 2013; Wiesman et al., 2016). Early studies suggested that the left IPC functions as a phonological store for verbal information (Baldo and Dronkers, 2006), however other studies have indicated that this region is also active during the memorization of visual and temporal-order information (Hayama et al., 2012; Majerus et al., 2007, 2006; Sneve et al., 2013). In fact, this region is now thought to be essential for the encoding of both item and item-order information in the visual and verbal domains, and maintains wide-ranging functional connections to support processing of this information (Majerus et al., 2007, 2006; Sneve et al., 2013). Taken together, this literature suggests that left inferior parietal regions might function as an integration point for item and item-order information in short-term memory. Our findings extend this interpretation: the progressive encoding of item-order information in the left IPC was significantly related to sequence memory performance, and was functionally decoupled from earlier, sensory-specific, visual networks. Similarly, the left STC is also known to be highly active during the encoding of both verbal and non-verbal stimuli into short-term memory stores (Heinrichs-Graham and Wilson, 2015; Keysers et al., 2005; Kwok and Macaluso, 2015; Leung et al., 2004; Proskovec et al., 2016, 2018; Raabe et al., 2013; Tallon-Baudry et al., 2004; Wiesman et al., 2016). However, the underlying function of this activity is likely substantially different from the nearby IPC, and is thought to be more essential for the encoding and polysensory integration of visual and auditory information (Noesselt et al., 2007; Stevenson and James, 2009; Venezia et al., 2017; Werner and Noppeney, 2010). It is possible that this region is useful for associating incoming visual information with novel verbal representations in an item-order memory task such as ours, but this hypothesis remains to be tested experimentally. Top down signals from left IFC also exert modulatory control over the IPC and STC during short-term memory encoding (Sneve et al., 2013; Wiesman et al., 2016), and thus this region is thought to bias the sensory and item-order encoding functions of the STC and IPC, respectively, resulting in a dynamic system of executive control over active short-term memory encoding.

Further supporting the importance of these alpha dynamics for the loading of stimulus information into short-term memory stores, single trial linear mixed-effects modeling revealed that the progressive increase in neural response amplitude with increasing encoding load in these regions (i.e., the trial-wise encoding slope) significantly covaried with memory performance. Perhaps most intuitively, this encoding slope exhibited a positive relationship with reaction time in the IPC, such that a stronger encoding load effect (i.e., a more negative slope) related to enhanced memory performance. This provides a direct link between the progressive loading of stimulus information into short-term memory stores, relying on left-lateralized patterns of alpha oscillations, and the subsequent recall of this information. The opposite effect on task performance was observed in the STC: more negative encoding slopes were associated with reduced memory performance. While this was unexpected, it is likely that the STC performs an inherently different function than the IFC and IPC in short-term memory, such as the integration of stimulus information with verbal representations of their order. Future research is certainly needed to continue to parse apart the functional sub-components of this system. In addition to tracking task performance, these progressive neural response slopes were also associated with neural activity during the memory maintenance phase of the task in all three brain regions. This further supports the conceptualization that these regions were essential for loading stimulus information into a cognitive store during encoding, as the progressive loading effect was directly related to the local strength of neural oscillatory responses needed to maintain this information for eventual retrieval.

Beyond these local relationships with neural activity during the maintenance phase and behavioral performance at retrieval, all three regions exhibited significant decreases in interregional functional connectivity with bilateral visual cortices during encoding. In other words, prior to the onset of the encoding stimuli, alpha oscillations in the left IFC and IPC were significantly more coherent with lateral occipital activity in the same frequency band, indicating shared patterns of active processing and/or inhibition (Bonnefond and Jensen, 2012; Jensen et al., 2002; Klimesch, 2012; Klimesch et al., 2007). However, at the onset of the encoding phase, this coherence decreased sharply, suggesting that the neural dynamics serving the active loading of stimulus information into short-term memory are functionally distinct from the brain systems that process lower-level visual item information. This decoupling, paired with the lack of significant increases in functional connectivity between these regions and any other location across the brain, provides essential evidence that such encoding processes are at least partially distinct from other brain systems with established sensory functions. It is even possible that this functional decoupling of these regions from early sensory processing serves a protective role for the stimulus sequence information that is being actively maintained and updated during encoding, however this hypothesis requires further validation. Supporting this conceptualization, alpha increases from baseline levels during the maintenance of short-term memories is one of the most replicated findings in the relevant literature (Bonnefond and Jensen, 2012; Embury et al., 2019; Heinrichs-Graham and Wilson, 2015; Jensen et al., 2002; Jensen and Mazaheri, 2010; Proskovec et al., 2016; Proskovec et al., 2019a; Tuladhar et al., 2007; Wiesman et al., 2016; Wilson et al., 2017), and is thought to represent the active inhibition of incoming visual input, in order to protect maintained information. Importantly, previous work has shown that this maintenance synchronization is spatially limited to early visual regions, while more anterior regions concurrently exhibit a robust alpha desynchronization during short-term maintenance (Embury et al., 2019; Heinrichs-Graham and Wilson, 2015; Proskovec et al., 2016; Proskovec et al., 2019a; Wiesman et al., 2016; Wilson et al., 2017), which aligns with our finding of left-lateralized desynchronizations in alpha activity in the current study.

Despite the potential novelty of our findings, it is also important to note the limitations of this work. First, it should be considered that, to some degree, the active loading of item information into memory and its concurrent maintenance in short-term stores are impossible to completely separate, as any multi-item set will necessarily be encoded sequentially and concurrently maintained. This said, although our paradigm is no exception to this rule, it still provides a unique and novel way to examine short-term memory load effects *under the context of active stimulus encoding*. This is particularly important, as deficits in encoding have been recognized in a number of neurological disorders (e.g., Baran et al., 2009; Bäckman et al., 2001; Golby et al., 2005; Kim et al., 2014; Lenartowicz et al., 2019; Rombouts et al., 2000; Wiesman et al., 2016; Woods et al., 2009). Our definition of the spectral and spatial features of the population-level neural activity underlying this function might serve as a starting point for future research targeting these deficits using frequency-targeted, non-invasive brain stimulation methods (Helfrich et al., 2014; Thut et al., 2011; Wiesman et al., 2018b) or steady-state sensory stimuli (Spaak et al., 2014; Wiesman et al., 2018a). Second, though evidence for lateralization of neural memory systems due to handedness is sparse, the bias in our participant sample towards right-handed individuals makes it impossible to exclude this as a potential reason for the lateralization of our alpha findings. Future studies should investigate such a prospect. Third, although beamforming has numerous benefits as a source-imaging method, the necessity of matrix regularization to compensate for rank deficiency introduced by tSSS in this approach has the potential to introduce a mild spatial bias. However, this bias would be consistent across the contrast of interest in this study (i.e., the three sequential stimulus items), and thus the resulting interpretations would largely be the same. Finally, although there are a number of benefits to using a single-trial approach to modeling relationships between neural dynamics and task performance, this method inherently reduces the use of task performance metrics to those that can be modeled at the level of each trial. In many cases, difference metrics, computed as a contrast between correct and incorrect trials, have been suggested as a way to null interceding effects of no interest (e.g., motor function), but such contrast metrics are not amenable to single trial models, and thus were not considered here. Even in light of these limitations, further studies of this kind will almost certainly contribute to a more thorough understanding of the dynamic and sophisticated distributed processing serving short-term memories in the human brain.

## Supplementary Material

1
