# Supplementary material for: Stairway to memory: Left-hemispheric alpha dynamics index the progressive loading of items into a short-term store
Source: Neuroimage. Author manuscript; Available in PMC 2021 Aug 10. (PMC8354033; doi:10.1016/j.neuroimage.2021.118024)
Supplement: 1 [file NIHMS1715924-supplement-1.pdf]

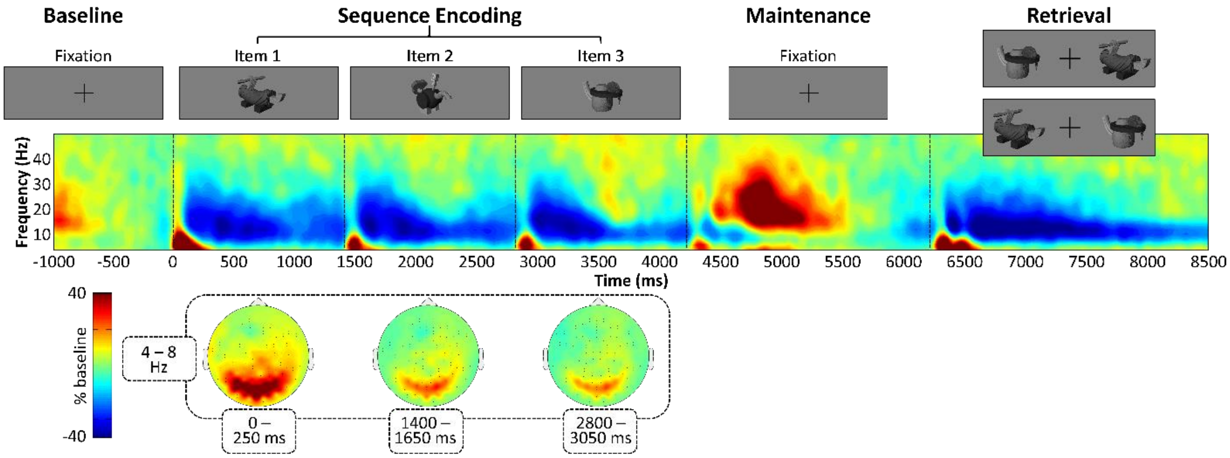

Figure S1. Sensor-level responses in the theta band. Oscillatory neural responses to the memory task (top) are displayed from a representative sensor (middle; MEG1923). Significant responses from baseline in the theta band (4 – 8 Hz) are plotted topographically (bottom) for each of the three sequence stimuli. The shared colorbar indicates percent change from baseline for both the spectrogram and topographic data.

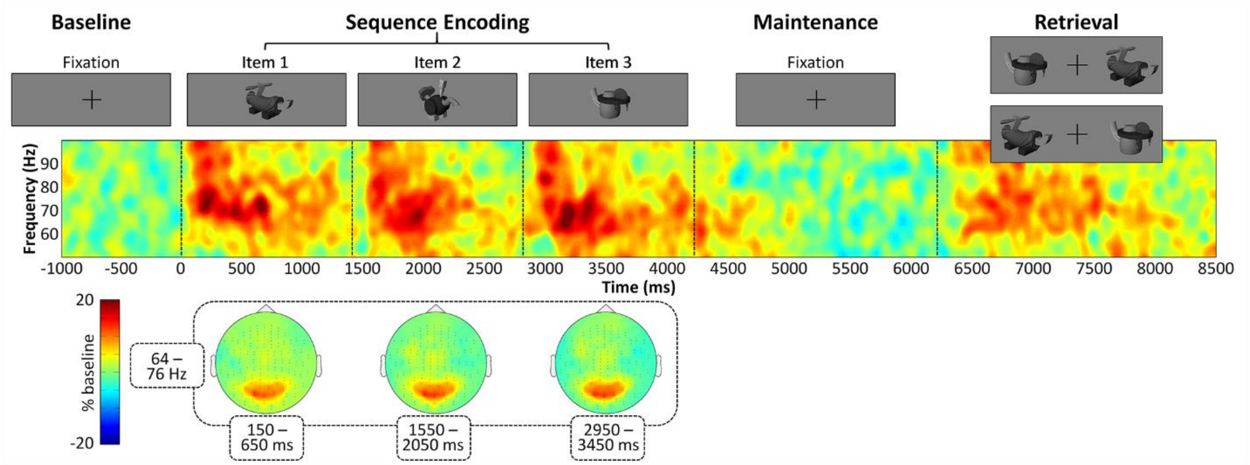

Figure S2. Sensor-level responses in the gamma band. Oscillatory neural responses to the memory task (top) are displayed from a representative sensor (middle; MEG1923). Significant responses from baseline in the gamma band (64 – 76 Hz) are plotted topographically (bottom) for each of the three sequence stimuli. The shared colorbar indicates percent change from baseline for both the spectrogram and topographic data.

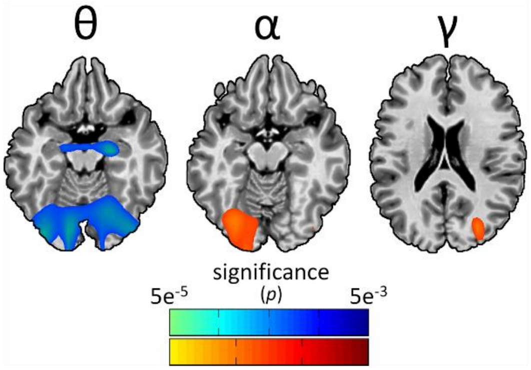

Figure S3. Supplementary whole-brain response slope analysis results. Statistical maps represent the clusters exhibiting significant response slopes after first-level voxel-wise thresholding ($p < .005$), per each oscillatory response. Importantly, only the theta slope cluster (left) was significant after second-level correction for mulitple comparisons (FWE cluster correction in SPM12).

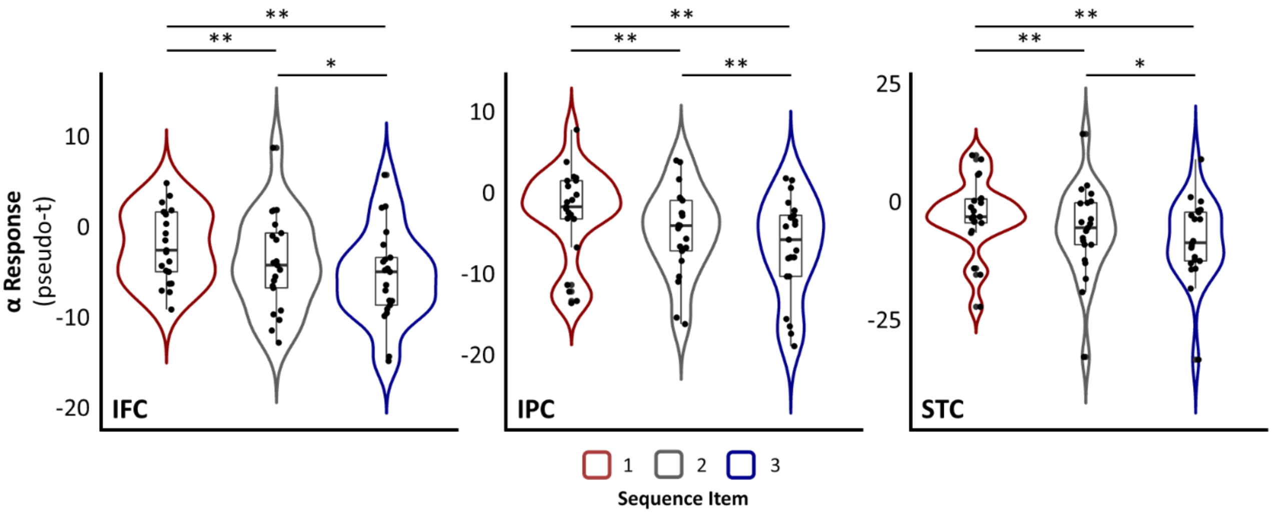

Figure S4. Post-hoc testing of alpha encoding slope data. Post-hoc testing of alpha response amplitudes at each of the three left-lateralized peaks indicated a significant stepwise decrease as a function of sequence progression. Sequence item is indicated by the color of each violin plot, and asterisks above indicate significance after correction for multiple comparisons. Tests are corrected within each region using the Holm-Bonferroni method. ***p < .01, corrected. *p < .05, corrected.

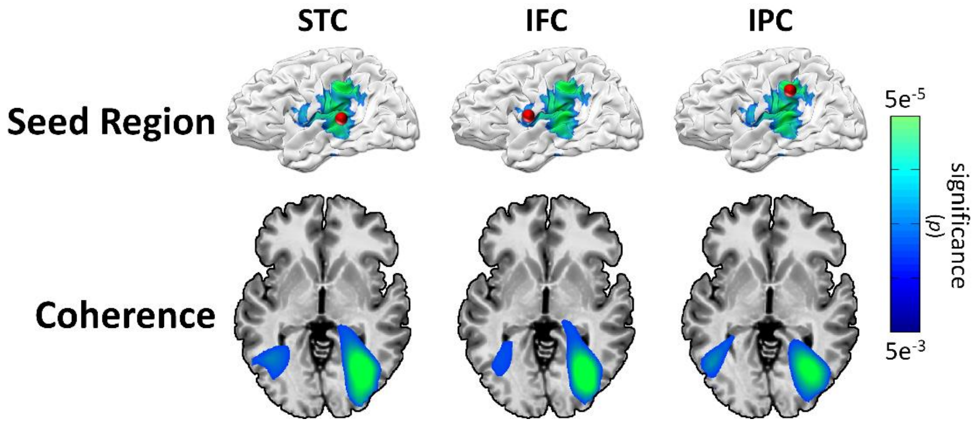

Figure S5. Left-hemisphere alpha memory store dynamics are functionally distinct from early visual networks. Statistical maps on the bottom represent significant whole-brain coherence clusters ($p < .005$; $k > 500$ voxels), above and beyond the effects of amplitude at the respective seed region (indicated above) and at each voxel. Cool colors indicate a significant decrease from baseline levels of coherence (i.e., functional connectivity).

Table S1. Linear mixed effects model of regional alpha encoding slopes on sequence memory performance.

Fixed Effects[a]	Estimate	*SE*	*df*	*t*	*p*
(Intercept)	1325.13	36.84	1938	35.97	<.001
IFC-slope	4.11	3.76	1938	1.09	.2749
IPC-slope	17.73	6.85	1938	2.59	.0097
STC-slope	-28.11	11.04	1938	-2.55	.0110

a. Dependent variable: reaction time. IFC: inferior frontal cortex; IPC: inferior parietal cortex; STC: superior temporal cortex.

Random Effects	Intercept	Residual
Participant	164.91	-
Participant/Trial	315.10	122.18

Table S2. Linear mixed effects model of inferior frontal alpha encoding slopes on maintenance neural responses.

Fixed Effects[a]	Estimate	*SE*	*df*	*t*	*p*
(Intercept)	0.03	0.02	1940	1.76	.0791
IFC-slope	0.01	0.00	1940	3.31	.0009

a. Dependent variable: alpha-frequency (10 – 20 Hz) neural response during maintenance. IFC: inferior frontal cortex.

Random Effects	Intercept	Residual
Participant	0.08	-
Participant/Trial	0.29	0.11

Table S3. Linear mixed effects model of inferior parietal alpha encoding slopes on maintenance neural responses.

Fixed Effects[a]	Estimate	*SE*	*df*	*t*	*p*
(Intercept)	0.05	0.03	1940	1.55	.1213
IPC-slope	0.02	0.00	1940	3.56	.0004

a. Dependent variable: alpha-frequency (10 – 20 Hz) neural response during maintenance. IPC: inferior frontal cortex.

Random Effects	Intercept	Residual
Participant	0.13	-
Participant/Trial	0.37	0.14

Table S4. Linear mixed effects model of superior temporal alpha encoding slopes on maintenance neural responses.

Fixed Effects[a]	Estimate	*SE*	*df*	*t*	*p*
(Intercept)	0.02	0.02	1940	0.85	.3954
STC-slope	0.01	0.01	1940	2.36	.0185

a. Dependent variable: alpha-frequency (10 – 20 Hz) neural response during maintenance. STC: superior temporal cortex.

Random Effects	Intercept	Residual
Participant	0.09	-
Participant/Trial	0.32	0.12
